# Fungal social barriers: to fuse, or not to fuse, that is the question

**DOI:** 10.1080/19420889.2020.1740554

**Published:** 2020-03-16

**Authors:** A. Pedro Gonçalves, N. Louise Glass

**Affiliations:** a Department of Plant and Microbial Biology, University of California, Berkeley, CA, USA; b Environmental Genomics and Systems Biology Division, Lawrence Berkeley National Laboratory, Berkeley, CA, USA

**Keywords:** Cell-cell fusion, allorecognition, intercellular cooperation, multicellularity

## Abstract

Cell fusion takes place in all domains of life and contributes greatly to the formation of complex multicellular structures. In particular, many fungi, such as the filamentous *Neurospora crassa*, rely on conspecific somatic cell fusion to drive the unicellular-to-multicellular transition and formation of the interconnected mycelial syncytium. This can, however, lead to the transmission of infectious elements and deleterious genotypes that have a negative impact on the organismal fitness. Accumulating evidence obtained from natural populations suggests that *N. crassa* has evolved various self/non-self or allorecognition systems to avoid fusion between genetically non-identical spores or hyphae at all costs. Here we present an overview of the recent advances made in the field of fungal allorecognition, describe its genetic basis, and comment on its evolutionary meaning. These data pinpoint the multilayered complexity of the cooperative social behaviors undertaken by a model eukaryotic microbe.

## Cell-cell fusion: a social lifestyle

The classical philosopher Aristotle once wrote that “Man is by nature a social animal” (*ca*. 328 BC). That is not less true at the cellular level, where social behaviors are typified by the process of cell-cell fusion. Cell fusion occurs in all domains of life and all humans originate from a cell fusion event – an oocyte and a sperm cell fuse to form a zygote and the germinal stage of development ensues. More generally, cell fusion is involved in various stages of development such as syncytiotrophoblast and skeletal muscle formation, bone remodeling, organ sculpting, neuronal development, and tumorigenesis, just to enumerate a few (recently reviewed by [1,2]). In fungi, more specifically in the eukaryotic model *Neurospora crassa*, somatic cell fusion drives the unicellular-to-multicellular transition and is fundamental for the formation of the interconnected hyphal network known as the mycelium [3].

### Cell fusion between genetically non-identical *N. crassa* cells is controlled by allorecognition systems

The mycelial colony constitutes a polarized syncytium that expands and radiates as a result of tip elongation and cooperative cell fusion, allowing maximal ability to colonize substrates [4]. However, this example of conspecific cooperation comes with a risk. On the one hand, Cell-cell and hyphal fusion within and between colonies potentially enhances the ability to withstand environmental variations and promotes an increase in organismal size due to the public nature of the created goods in a multinuclear syncytium. However, the transition to a multicellular state can result in conflict due to the potential transmission of infectious elements via somatic fusion events and incorporation of deleterious mitochondrial or nuclear genotypes that are detrimental for the overall fitness of the individuals. In this context, evidence from *Neurospora* and other microbes has shown that the higher the genetic relatedness, the higher the likelihood that cells will undergo cooperative behaviors in order to prevent parasitism and the exploitation of public goods by cheaters, defined as selfish units that receive benefit at the cost of others [5–9].

In *N. crassa*, the initial stages of multicellular colony establishment are characterized by cell fusion events between germinated unicellular spores (germlings) that are in close proximity. In natural settings, spores encounter numerous other spores with varying degrees of genetic similarity and fusion of genetically dissimilar cells results in cytoplasmic pools that contain a mixture of organelles originating from dissimilar genetic backgrounds, the so-called ‘heterokaryons’. To reduce the risk of detrimental heterokaryon formation, fungi have evolved allorecognition systems that ‘surveil’ genetic identity and impede the process of cell fusion from occurring whenever two partner cells are genetically distant. A well-established example of such a system is triggered during fusion of vegetative hyphae of *N. crassa*: if fusion between hyphae that are genetically identical at all *het* loci, a stable heterokaryon is formed; if hyphae have alternate specificity at any *het* locus, a defense response is elicited leading to cell death. Due to septal plugging, the death reaction is restricted to the heterokaryotic fusion cell and surrounding compartments, sparing the remainder of the colony [10]. This phenomenon, termed heterokaryon incompatibility or vegetative incompatibility, is put in place to prevent somatic parasitism and the transmission of infectious elements and mycoviruses [11–13]. Importantly, *het* loci typically display features of rapid diversification [14]. All *het* loci have been identified in hyphal-based experimental setups and *het*-gene-based allorecognition has not been extensively studied in germlings. Evidence exists that vegetatively incompatible strains can fuse at the germling stage in *Colletotrichum lindemuthianum* [15], while in *N. crassa*, germling fusion occurs, although delayed, in situations of heterokaryon incompatibility [16].

**Figure 1.**
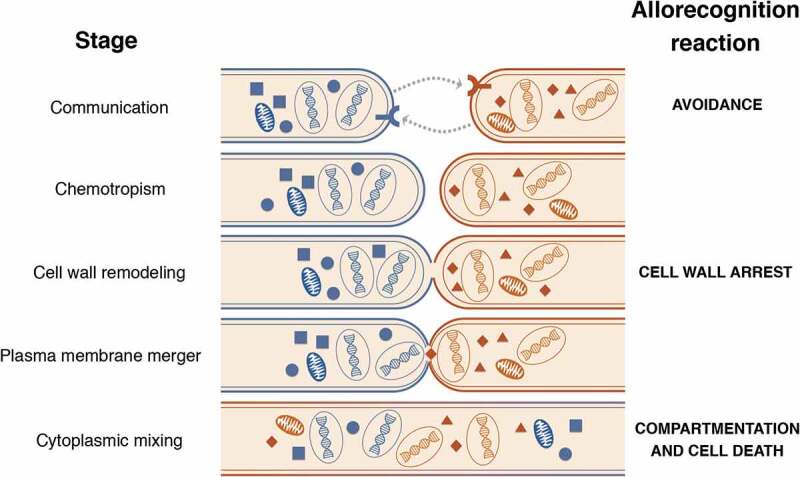
The different stages of somatic cell fusion in *N. crassa* and respective allorecognition checkpoint systems and genetic determinants that have been identified thus far. Genetic dissimilarity is represented by the two different colors.

More recently, our group has used population genomics to discover cell fusion-associated allorecognition checkpoints that occur in germlings as well as hyphae. Conceptually speaking, cell fusion in germlings and hyphae can be divided into four steps (Figure 1): 1) intercellular communication leading to chemotropic growth of the two fusing partners; 2) cell wall remodeling that is triggered after cell-cell contact has occurred; 3) plasma membrane merger; and 4) cytoplasmic mixing and the conclusion of the fusion process [17]. Cells that have identical allelic specificity at the determinant of communication loci (*doc-1* and *doc-2*) loci are capable of communicating with each other, while cells encoding *doc* alleles of different specificities fail to reinforce chemotropic interactions and consequently show a greatly reduced level of cell fusion [18]. The DOC-1 and DOC-2 loci encode proteins lacking annotation or conserved domains and therefore their biochemical function remains unclear. Cells with identical specificity at the *doc* loci will undergo chemotropic interactions, but a second cell fusion checkpoint can be triggered upon cell contact and adhesion. In this case, the ability to proceed with cell wall dissolution is defined by genetic identity at the cell wall remodeling loci (*cwr-1* and *cwr-2*). If cells have different *cwr* allelic specificity, adhered cells are unable to switch from chemotropism to cell wall dissolution, as shown by the continued oscillation of chemotropism-associated proteins and accumulation of cell wall material at the zone of contact [19]. CWR-1 is a carbohydrate-binding polysaccharide monooxygenase potentially involved in directly modifying the cell wall while CWR-2 is a poorly conserved protein with several transmembrane domains and thus hypothesized to behave as a receptor at the cell periphery.

When the *doc* and *cwr* checkpoints are crossed, cell fusion and cytoplasmic mixing occur with the cellular constituents becoming shared goods in cytoplasmic pools. However, a third checkpoint controlled by allelic specificity at the *sec-9* and *plp-1* loci regulates the post-fusion viability of heterokaryons in germlings and hyphae. If cells encode different allelic specificities at *sec-9*/*plp-1*, rapid vacuolization and cell death are triggered [20]. PLP-1 functions as a nucleotide-binding domain leucine-rich repeat containing receptor (NLR) in one cell, which is activated by interactions with the soluble N-ethylmaleimide sensitive factor attachment protein receptor (SNARE) SEC-9 in the fusion partner cell. More recently, another cell death-triggering locus was identified in wild *N. crassa* populations and that causes a phenotype similar to the *sec-9*/*plp-1* reaction; in this case, the allorecognition determinant is the *rcd-1* [21]. The *rcd-1* gene belongs to a large and uncharacterized family widespread in Ascomycota fungi and is one of the most polymorphic genes in the genomes of wild *N. crassa* and *Neurospora discreta* isolates (but not *Neurospora tetrasperma*) [21]. The evolutionary benefit and/or meaning of the consequences of activation of these cell death-inducing allorecognition systems are intriguing – as they were found in germlings, cell death results in organismal death. However, as with genetic differences at *het* loci, differences at *sec-9/plp-1* and *rcd-1* also cause programmed cell death of hyphal fusion compartments [20].

For all three germling fusion checkpoints, the corresponding alleles are highly polymorphic, segregate into discrete haplotypes, and show trans-species polymorphisms and evidence of balancing selecting acting on these loci. A simple calculation that takes into account the three germling checkpoints, the *het* hyphal checkpoints, and the number of haplotypes identified for each of them results in an estimated 2,211,840 incompatibility groups, that is, sets of strains that can only fuse successfully amongst themselves (Table 1). We speculate that it is highly possible that the addition of more isolates from various geographic provenances would result in the discovery of several more haplotypes, particularly in the case of the *het* loci, for which, in some cases, only two incompatible haplogroups have been described. Dozens of additional *het* loci have been recently identified [22] and experimental testing of their role in heterokaryon incompatibility will likely expand the number of self/non-self recognition systems. Moreover, we have preliminary evidence that additional allorecognition checkpoints are activated during germling cell fusion.

**Table 1.** Summary of allorecognition systems in *N. crassa*. The right column shows the cumulative calculation of the total number of incompatible groups identified so far. The estimated total of incompatible genotypes is indicated in bold.

Asexual stage	Cell-cell fusion step	Allorecognition reaction	Loci	References	Number of haplogroups	Cumulative number of incompatible genotypes
Germling and hyphal	Intercellular communication and chemotropism	Avoidance at distance	*doc*	[18]	5	5
	Cell wall remodeling	Arrest and cell wall accumulation upon contact	*cwr*	[19]	6	30 (5 x 6)
	Cytoplasmic mixing	Cell death	*sec-9/plp-1*	[20]	4	120 (30 x 4)
			*rcd-1*	[21]	2	240 (120 x 2)
Hyphal (*het* loci)	Cytoplasmic mixing	Cell death	*het-e*	[22]	3	720 (240 x 3)
			*het-c/pin-c*	[24]	3	2160 (720 x 3)
			*mat/tol*	[25]	2	4320 (2,160 x 2)
			9 additional *het* loci	[24,26–28]	2^9 *^	**2,211,840** (4320 x 2^9^)

* Two haplotypes were conservatively assumed since a systematic analysis of wild populations has not been performed.

A number of evolutionary pressures might have contributed to the high number of allorecognition systems in *N. crassa*. First of all, despite being important for multicellularity, cell fusion also provides an opportunity for cheater individuals to take advantage of shared cellular goods without participating in their production, weakening the fitness of the fungal colony [6]. As a consequence, multicellular cooperation is enhanced in highly related individuals ^6^. Secondly, allorecognition presents an effective mechanism to prevent the accumulation of mycoviruses, as shown in *Cryphonectria parasitica* [13], or mitochondrial defects, as shown in *N. crassa* [12] and *Neurospora intermedia* [23]. It seems puzzling that such a large number of incompatibility groups have been selected during evolution only for the purpose of avoidance of conspecific cell fusion, and therefore, additional research is required to fully comprehend the range of functions of these genetic identity-conferring molecules.

## Avoiding fusion at all costs

Taken together, our findings strongly suggest that *N. crassa* has evolved to avoid conspecific somatic cell fusion with genetically non-identical cells at all costs. This has likely been driven by the detrimental effects caused by the incorporation of cheaters and infectious elements into the mycelium, which in turn impairs organismal fitness [6,11–13,17]. Pre-fusion allorecognition (based on *doc* and *cwr* loci) could function as a tool to avoid the drastic cost of the downstream cell death-associated non-self recognition systems.

Altogether, these studies have shown a series of multilayered and intricate non-self recognition systems that surveil genetic identity whenever cell fusion is imminent (Figure 1). This phenomenon is a good illustration of the high level of complexity of the social interactions that can take place in the microbial world by seemingly simple organisms.
